# Laser-Machined Microcavities for Simultaneous Measurement of High-Temperature and High-Pressure

**DOI:** 10.3390/s140814330

**Published:** 2014-08-07

**Authors:** Zengling Ran, Shan Liu, Qin Liu, Ya Huang, Haihong Bao, Yanjun Wang, Shucheng Luo, Huiqin Yang, Yunjiang Rao

**Affiliations:** Key Laboratory of Optical Fiber Sensing and Communications (Ministry of Education of China), University of Electronic Science and Technology of China, Chengdu 611731, China; E-Mails: liushan0702@126.com (S.Li.); huangya500227@163.com (Y.H.); edwardbao@sina.cn (H.B.); wangyanjun8801@163.com (Y.W.); luoshucheng2008@126.com (S.Lu.); yanghq66@163.com (H.Y.); yjrao@uestc.edu.cn (Y.R)

**Keywords:** high pressure sensing, high temperature sensing, laser micromachining, micro-cavity, simultaneous measurement

## Abstract

Laser-machined microcavities for simultaneous measurement of high-temperature and high-pressure are demonstrated. These two cascaded microcavities are an air cavity and a composite cavity including a section of fiber and an air cavity. They are both placed into a pressure chamber inside a furnace to perform simultaneous pressure and high-temperature tests. The thermal and pressure coefficients of the short air cavity are ∼0.0779 nm/°C and ∼1.14 nm/MPa, respectively. The thermal and pressure coefficients of the composite cavity are ∼32.3 nm/°C and ∼24.4 nm/MPa, respectively. The sensor could be used to separate temperature and pressure due to their different thermal and pressure coefficients. The excellent feature of such a sensor head is that it can withstand high temperatures of up to 400 °C and achieve precise measurement of high-pressure under high temperature conditions.

## 1. Introduction

Fiber optic high-pressure sensors have been widely used in variety of industries. The in-fiber Bragg grating (FBG) pressure sensor and fiber optic Fabry-Perot (F-P) pressure sensor are the main types of fiber optic pressure sensors [1–3]. The pressure sensor fabrication techniques involve anodic bonding [4], wet etching [5,6], adhesive bonding [7], and sol gel bonding, *etc.*, however, they cannot be safely operated under high temperature conditions. In order to achieve accurate measurements, temperature discrimination is needed, such as the discrimination against temperature and strain [8]. As for accurate pressure measurement, a temperature sensor should be used to compensate the parameter shift induced by temperature variation near the pressure probe, especially for FBG pressure sensors. Particularly, the pressure sensor value shifts caused by temperature variation are much more serious under high temperature [6,9]. At this time, temperature compensation is very essential for almost any pressure sensor due to the large temperature variation range [10,11]. As we know, precise pressure measurement is quite important for many high-temperature applications, such as health monitoring of engines and down-hole oil exploration [12–15]. However, both the FBG written by UV laser exposure and the extrinsic Fabry-Perot interferometric (EFPI) sensor formed by inserting two optical fibers into a glass capillary manually cannot withstand high temperatures. Wang *et al.* proposed a type of all silica Fabry-Pérot (F-P) sensor for the measurement of pressure under high temperature conditions [16], which is fabricated by sandwiching a short-hollow-core fiber between two lengths of single-mode fiber. Although good high-temperature stability was obtained, its manual assembly is time-consuming [15]. By 157 nm laser-micromachining, cascaded micro Fabry-Perot (F-P) cavities have been proposed and demonstrated for simultaneous measurement of high temperature and strain, or high temperature and refractive index [17].

In this paper, a 157 nm laser-machined sensor with cascaded microcavities is proposed for simultaneous measurement of high-temperature and pressure. The sensor head is an all fiber, self-enclosed compact device, which overcomes the long-lasting problem of the conventional EFPI sensor, namely that the bonding strength is not good enough or the bonding adhesive could age quickly at high temperatures. Furthermore, its dual parameter acquisition capability provides an effective solution for precise measurement of high pressure under high temperature.

## 2. Sensors Structure and Fabrication

**Figure 1.**
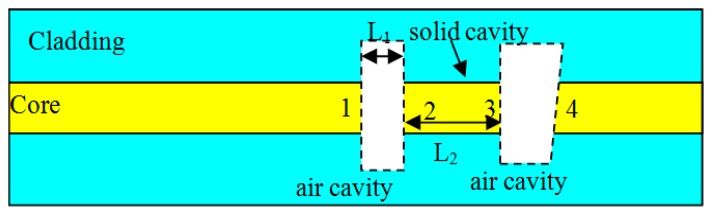
Structure of the sensor head, showing an in-fiber intrinsic F-P cavity and two air cavities, where the numbers “1”, “2”, “3” and “4” label are the four reflection surfaces in the structure.

The structure of the sensor head, which contains two extrinsic in-fiber air cavities and an intrinsic solid F-P cavity, is shown schematically in Figure 1. The fiber end is supposed to be sealed in the fluid or gas whose temperature and pressure are to be measured.

As shown in Figure 1, there are four interfaces in the sensor head, labeled as “1”, “2”, “3” and “4”, respectively. Interfaces 1 and 2 form a short cavity, denoted as cavity 1, while interfaces 1 and 3 form another cavity, denoted as cavity 2. Cavity 3 is formed by interfaces 2 and 3. Cavity 4 is formed by interfaces 4 and 3. The tilt interface 4 has very low reflectivity, so the tilt interface reduces the back-reflection toward the interface 3. Cavity 1 and cavity 2 are used as sensing elements. The lengths of cavity 1, cavity 2, and cavity 3 are L_1_, L_1_ + L_2_ and L_2_, respectively.

**Figure 2.**
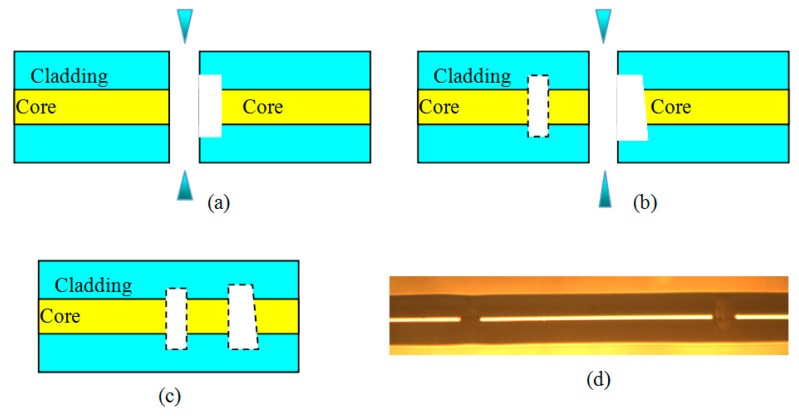
Fabrication and configuration of high temperature and high pressure sensor (**a**) fabrication the first air cavity; (**b**) fabrication the second air cavity; (**c**) configuration of sensor; (**d**) photograph of sensor from splicer.

As shown in Figure 2, to fabricate the sensor head, we first produced a circular hole with a depth of ∼20 μm and a diameter of ∼60 μm at the center of the cross section of a single-mode fiber by using a 157-nm laser micromachining system, which consisted of a 157-nm pulsed laser (LPF202, Coherent, Santa Clara, CA, USA), an optical focusing system (with 25 times demagnification), an observing system, and a precision translation stage.

**Figure 3.**
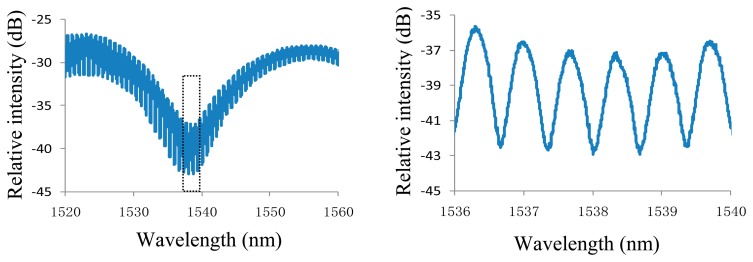
(**a**) Measured reflected spectrum of the sensor; (**b**) Close-up display of the fringe.

The pulse energy density, pulse width, and pulse repetition rate used were 20 J/cm^2^, 15 ns, and 20 Hz, respectively. We used 100 pulses to produce the hole, which took only 5 s to complete. The hole was larger than the core but smaller than the cladding, as shown in Figure 2a. After then, splicing the fiber with the hole and a cleaved fiber end to enclose the hole, an air F-P cavity was formed with a cavity length of ∼35 μm. Next, we cleaved the fiber at a short distance of ∼1180 μm from the air cavity, and spliced the cleaved fiber end to a circular hole to form another air cavity to protect the third interface, which cavity length is ∼50 μm. To reduce the reflectivity of interface 4, the bottom surface of the circular hole is made into an angular surface, which is fabricated by adjusting the angle between 157 nm laser beam and the fiber end face to a non-vertical state. A microscopic image of the fabricated sensor head is shown in Figure 2d. The reflected spectrum of the sensor is measured and shown in Figure 3. It is can be seen that there are narrow and broad fringes which are due to long and short air cavity (cavity 2 and cavity 1), respectively.

## 3. Sensing Principle

The sensor head in Figure 2d is made of full silica fiber, which gives excellent high temperature stability. Since these two cavities (cavity 1 and 2) have different cavity lengths and configurations, they should have different responses under a certain variation of pressure or temperature. Using this sensor head to simultaneously measure high-temperature and high-pressure is based on the following equations [18]:

(1) $$\Delta OPD_{1}=\Delta OPD_{1}(\Delta T)+\Delta OPD_{1}(\Delta P)=\alpha_{1}\cdot \Delta T+\beta_{1}\cdot \Delta P$$

(2) $$\Delta OPD_{2}=\Delta OPD_{2}(\Delta T)+\Delta OPD_{2}(\Delta P)=\alpha_{2}\cdot \Delta T+\beta_{2}\cdot \Delta P$$

where *α_1_* and *α_2_* are the thermal coefficients of cavity 1 and 2 as shown in Figure 1, and *β_1_* and *β_2_* are pressure coefficients of cavity 1 and 2, and ΔOPD_1_ and ΔOPD_2_ are the optical path differences (OPD) of cavity 1 and 2, and ΔT and ΔP are the value of temperature and pressure variations. Cavity 1, it is an air cavity, whose OPD variation under pressure is given by:

(3) $$\Delta OPD_{1}(\Delta P)=2•\Delta L=\beta_{1}•\Delta P=2\frac{L_{1}(1-2\mu )R_{0}^{2}}{E(R_{0}^{2}-R^{2})}\Delta P$$

where *R_0_* and *R* are the radius of the fiber and the radius of the air hole, respectively, *E* is Young's modulus, and *μ* is Poisson's ratio.

Cavity 2, it is composed of an air cavity and an intrinsic F-P cavity, whose OPD variation under pressure is given by [19]:

(4) $$\Delta OPD_{2}(\Delta P)=\beta_{2}\cdot \Delta P=2\left[\frac{L_{1}(1-2\mu )R_{0}^{2}}{E(R_{0}^{2}-R^{2})}+\frac{nL_{2}(1-2\mu )}{E}+\alpha_{\text{n}}nL_{2}\right]\Delta P$$

where $\alpha_{\text{n}}=-\frac{n^{2}(1-2\mu )[P_{12}-\mu (P_{11}+P_{12})]}{2E}$, *α*_n_ is the refractive index variation coefficient induced by pressure. The OPD variation of a fiber-optic F-P cavity induced by temperature variation, which is a linear relationship with temperature, has been extensively studied. If the thermal and pressure coefficients of cavity 1 and 2 are measured experimentally, the pressure and temperature could be clearly separated based on Equations (1) and (2).

## 4. Experiments

**Figure 4.**
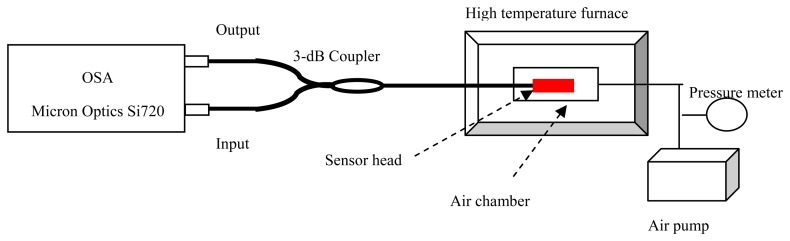
Experimental setup for high-temperature and high-pressure test.

In order to confirm the thermal and pressure coefficients sensor head in Figure 2d, the high-temperature and high-pressure response test is performed. The experimental setup of the sensor is shown in Figure 4.

**Figure 5.**
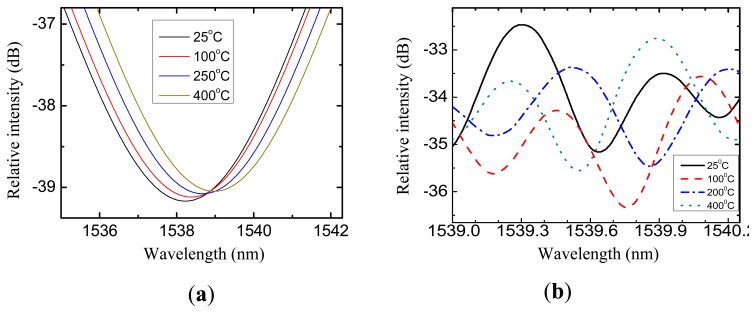
The spectrum shift of with temperature. (**a**) cavity 1 and (**b**) cavity 2.

In our experiments, the sensor head was sealed into an air chamber (CTS2008 pressure meter, with accuracy of 0.05%) and put into a high temperature furnace (Heson, HS-1100, Shanghai, China, from room temperature to 1050 °C, with accuracy of ±1 °C). An optical spectrum analyzer (OSA; Si720, Micron Optics, Atlanta, GA, USA, with a resolution of 0.25 pm) provided a swept light source for the sensor and at the same time measured the reflective spectrum of the sensor. During the tests, the temperature response of the sensor is measured, as given by Figure 5, where the fringes of the cavity 1 and 2 overlapped. In order to tracking the pit wavelength shift of cavity 1, the original reflected spectrum is filtered by a low pass filter, as illustrated by Figure 5a. The spectrum shift of cavity 2 is seen in Figure 5b.

**Figure 6.**
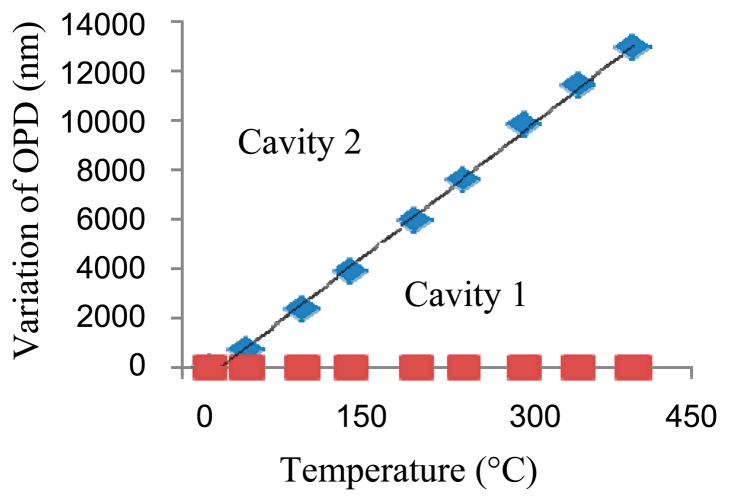
Variation of OPDs with temperature.

**Figure 7.**
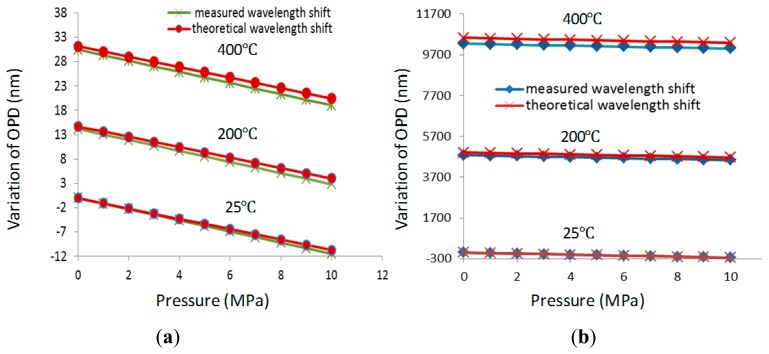
Variation of OPDs with pressure at different temperature (**a**) cavity 1 and (**b**) cavity 2.

The temperature responses of cavity 1 and 2 are tested from 25 °C to 400 °C, as shown in Figure 6. The tested thermal coefficients of cavity 1 and 2 are ∼0.0779 nm/°C and ∼32.3 nm/°C, respectively. The temperature of the furnace is set to three fixed temperatures of 25 °C, 200 °C and 400 °C, respectively, and then the pressure responses of cavity 1 and 2 are tested from 0 MPa to 10 Mpa, respectively, as shown in Figure 7. The pressure coefficients of cavity 1 and 2 are ∼1.14 nm/MPa and ∼24.6 nm/MPa, respectively. The temperature and pressure characteristics of the sensor show good linearity and agree well with the theoretical predictions. Based on the difference of their sensing coefficients, pressure and temperature could be simultaneously measured using Equations (1) and (2).

**Figure 8.**
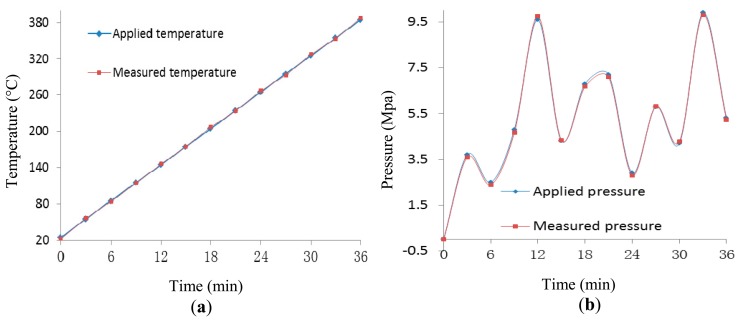
Experimental results of simultaneous measurement of temperature and pressure. (**a**) measurement of temperature; (**b**) measurement of pressure.

To investigate the capability of simultaneous measurement of temperature and pressure, the temperature was varied from room temperature to about 365 °C, at a rate of 10 °C/min increased by setting the high temperature furnace in the test apparatus. Meantime, the pressure was also measured over a range of 0 to10 MPa. At random, the temperature and pressure were recorded and compared as shown in Figure 8, showing good agreement between the measured pressure and the applied pressure.

## 5. Conclusions

In this paper, a 157 nm laser-machined sensor with cascaded microcavities is proposed, which could be used to separate temperature and pressure for their different thermal and pressure coefficients. It is shown that the sensor can withstand a temperature of up to 400 °C, and could be used to simultaneously measure high-temperature and high-pressure in the experiments. Such a sensor could find important applications in automotive and aerospace industry fields and down hole oil exploration, *etc*.

## References

[1] Jewart C.M., Wang Q., Canning J., Grobnic D., Mihailov S.J., Chen K.P. (2010). Ultrafast femtosecond-laser-induced fiber Bragg gratings in air-hole microstructured fibers for high-temperature pressure sensing. Opt. Lett..

[2] Chen T., Chen R., Jewart C., Zhang B., Cook K., Canning J., Chen K.P. (2011). Regenerated gratings in air-hole microstructured fibers for high-temperature pressure sensing. Opt. Lett..

[3] Ma J., Ju J., Jin L., Jin W. (2011). A Compact Fiber-Tip Micro-Cavity Sensor for High-Pressure Measurement. IEEE Photon. Technol. Lett..

[4] Zhou J., Dasgupta S., Kobayashi H., Wolff J.M., Jackson H.E., Boyd J.T. (2001). Optically interrogated MEMS pressure sensors for propulsion applications. Opt. Eng..

[5] Zhu Y., Wang A. (2005). Miniature fiber optic pressure sensor. IEEE Photon. Technol. Lett..

[6] Donlagic D., Cibula E. (2005). All-fibre high-sensitivity pressure sensor with SiO_2_ diaphragm. Opt. Lett..

[7] Yu B., Kim D.W., Deng J.D., Xiao H., Wang A. (2003). Fiber Fabry–Pérot sensors for detection of partial discharges in power transformers. Appl. Opt..

[8] Liu H.B., Liu H.Y., Peng G.D., Chu P.L. (2003). Strain and temperature sensor using a combination of polymer and silica fibre Bragg gratings. Opt. Commun..

[9] Wu C., Fu H.Y., Qureshi K.K., Guan B.O., Tam H.Y. (2011). High-pressure and high-temperature characteristics of a Fabry–Perot interferometer based on photonic crystal fiber. Opt. Lett..

[10] Wang W., Wu N., Tian Y., Niezrecki C., Wang X. (2010). Miniature all-silica optical fiber pressure sensor with an ultrathin uniform diaphragm. Opt. Express.

[11] Lee B. (2003). Review of the present status of optical fiber sensors. Opt. Fiber Technol..

[12] Goustouridis D., Normand P., Tsoukalas D. (1998). Ultraminiature silicon capacitive pressure-sensing elements obtained by silicon fusion bonding. Sens. Actuators A Phys..

[13] Schroeder R.J., Ramos R.T., Yamate T. (1999). Fiber optic sensors for oil field services. Proc. SPIE.

[14] Fu H.Y., Wu C., Tse M.L.V., Zhang L., Cheng K.C.D., Tam H.Y., Guan B.O., Lu C. (2010). High pressure sensor based on photonic crystal fiber for downhole application. Appl. Opt..

[15] Ran Z., Liu Z., Rao Y., Xu F., Sun D., Yu X., Xu B., Zhang J. (2011). Miniature Fiber-Optic Tip High Pressure Sensors Micromachined by 157 nm Laser. IEEE Sens. J..

[16] Wang X., Xu J., Zhu Y., Cooper K.L., Wang A. (2006). All-fused silica miniature optical fiber tip pressure sensor. Opt. Lett..

[17] Ran Z., Li C., Zuo H.M., Chen Y. (2013). Laser-Machined Cascaded Micro Cavities for Simultaneous Measurement of Dual Parameters under High Temperature. IEEE Sens. J..

[18] Singh H., Sirkis J.S. (1997). Simultaneously measuring temperature and strain using optical fiber microcavities. J. Lightwav. Technol..

[19] Zhang Y., Feng D., Liu Z., Guo Z., Dong X., Chiang K.S., Chu B.C.B. (2001). High-sensitivity pressure sensor using a shielded polymer-coated fiber Bragg grating. IEEE Photon. Technol. Lett..

